# Elevated levels of plasma symmetric dimethylarginine and increased arginase activity as potential indicators of cardiovascular comorbidity in rheumatoid arthritis

**DOI:** 10.1186/s13075-018-1616-x

**Published:** 2018-06-08

**Authors:** Unnikrishnan M. Chandrasekharan, Zeneng Wang, Yuping Wu, W. H. Wilson Tang, Stanley L. Hazen, Sihe Wang, M. Elaine Husni

**Affiliations:** 1 Department of Cellular and Molecular Medicine, Lerner Research Institute, Cleveland Clinic, Cleveland, OH USA; 2 Department of Mathematics, Cleveland State University, Cleveland, OH USA; 3 Department of Cardiovascular Disease, Heart and Vascular Institute, Cleveland Clinic, Cleveland, OH USA; 4 Department of Laboratory Medicine, Cleveland Clinic, Cleveland, OH USA; 5 Department of Rheumatic and Immunologic Diseases, Cleveland Clinic, Cleveland, OH USA

**Keywords:** Rheumatoid arthritis, l-arginine, Dimethylarginines, Arginase, Nitric oxide

## Abstract

**Background:**

Rheumatoid arthritis (RA) patients are at high risk of developing cardiovascular disease (CVD). In RA, chronic inflammation may lead to endothelial dysfunction, an early indicator of CVD, owing to diminished nitric oxide (NO) production. Because l-arginine is the sole precursor of NO, we hypothesized that levels of l-arginine metabolic products reflecting NO metabolism are altered in patients with RA.

**Methods:**

Plasma samples from patients with RA (*n* = 119) and age- and sex-matched control subjects (*n* = 238) were used for this study. Using LC-MS/MS, we measured plasma levels of free l-arginine, l-ornithine, l-citrulline, l-*N*^G^-monomethyl arginine (MMA), asymmetric dimethylarginine (ADMA), and symmetric dimethylarginine (SDMA). We compared global arginine bioavailability ratio (GABR) (i.e., ratio of l-arginine to l-ornithine + l-citrulline) and arginine methylation index (ArgMI) (i.e., ADMA + SDMA/MMA) in patients with RA vs. control subjects. Plasma arginase activity was measured using a sensitive arginase assay kit. The relationship of l-arginine metabolites and arginase activity to CVD risk factors was evaluated using Pearson’s chi-square test.

**Results:**

Compared with healthy control subjects, the RA cohort showed significantly lower levels of plasma l-arginine (46.11 ± 17.29 vs. 74.2 ± 22.53 μmol/L, *p* < 0.001) and GABR (0.36 ± 0.16 vs. 0.73 ± 0.24, *p* < 0.001), elevated levels of ADMA (0.76 ± 0.12 vs. 0.62 ± 0.12 μmol/L, *p* < 0.001), SDMA (0.54 ± 0.14 vs. 0.47 ± 0.13 μmol/L, *p* < 0.001), and ArgMI (6.51 ± 1.86 vs. 5.54 ± 1.51, *p* < 0.001). We found an approximately fourfold increase in arginase activity (33.8 ± 1.1 vs. 8.4 ± 0.8 U/L, *p* < 0.001), as well as elevated levels of arginase-mediated l-arginine catalytic product l-ornithine (108.64 ± 30.26 vs. 69.3 ± 20.71 μmol/L, *p* < 0.001), whereas a nitric oxide synthase (NOS) catalytic product, the l-citrulline level, was diminished in RA (30.32 ± 9.93 vs. 36.17 ± 11.64 μmol/L, *p* < 0.001). Patients with RA with existing CVD had higher arginase activity than patients with RA without CVD (*p* = 0.048).

**Conclusions:**

Global l-arginine bioavailability was diminished, whereas plasma arginase activity, ADMA, and SDMA levels were elevated, in patients with RA compared with healthy control subjects. Plasma SDMA was associated with hypertension and hyperlipidemia in patients with RA. This dysregulated l-arginine metabolism may function as a potential indicator of CVD risk in patients with RA.

## Background

**Fig. 1:**
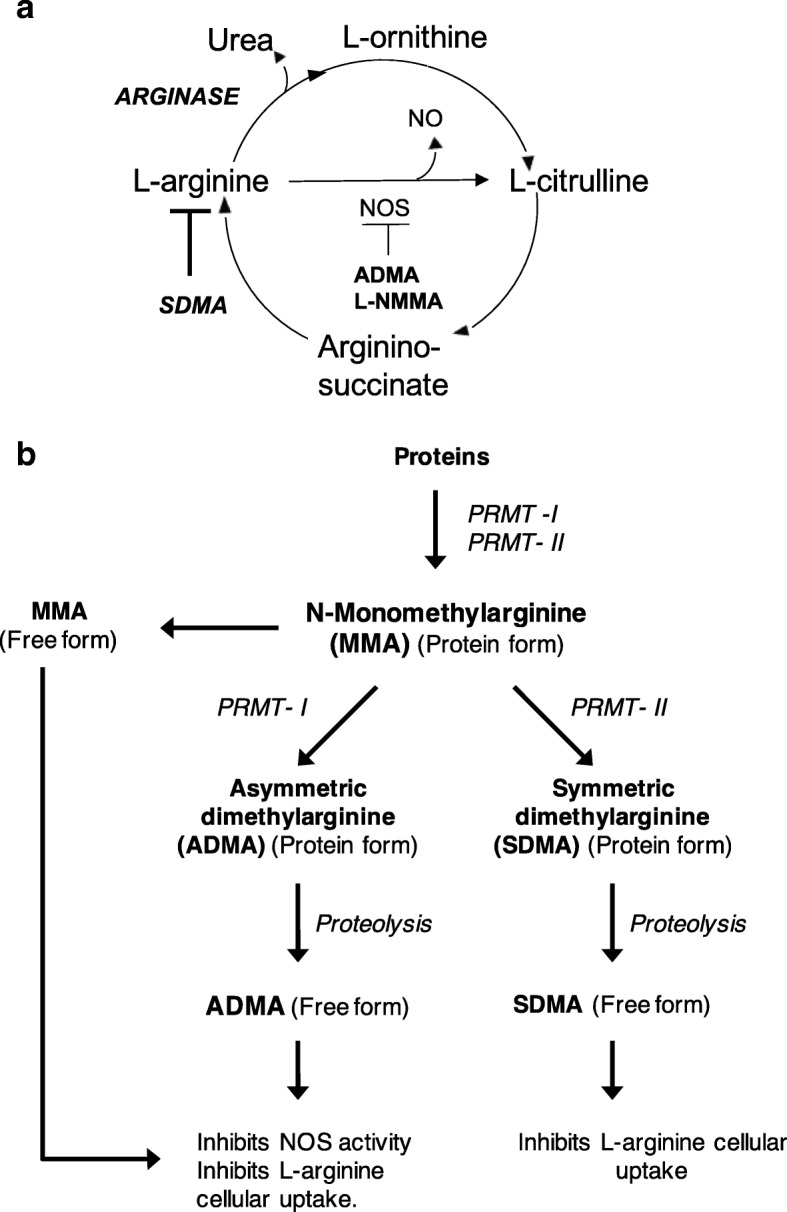
Urea cycle and dimethylarginine metabolic pathways. **a** l-Arginine is the common substrate of arginase and nitric oxide synthase (NOS). Asymmetric dimethylarginine (ADMA) and l-*N*^G^-monomethyl arginine (MMA) are potent inhibitors of NOS, whereas symmetric dimethylarginine (SDMA) inhibits nitric oxide (NO) production by inhibiting l-arginine cellular uptake. **b** Schematic diagram showing the pathways that produce methylated arginines (MMA, ADMA, and SDMA). *PRMT* Protein arginine methyltransferase

Rheumatoid arthritis (RA) affects approximately 0.5–1% of the U.S. general adult population [1–3]. Patients with RA have both articular and extraarticular manifestations, such as accelerated cardiovascular disease (CVD), which accounts for up to 50% of the deaths in this population [4, 5]. The cardiovascular morbidity and mortality are hypothesized to be due in part to persistent systemic inflammation; however, the exact mechanisms remain undetermined. Unfortunately, traditional cardiac risk factors seen in the normal population do not completely account for this increase in CVD in RA, a prototypical rheumatic disease [6, 7]. There is a great unmet need to identify nontraditional molecular biomarkers and related pathways responsible for the higher CVD incidence in patients with RA.

l-arginine is the common substrate of nitric oxide synthase (NOS) and arginases [8]. NOS catalyzes l-arginine to generate nitric oxide (NO) and l-citrulline, whereas arginases catalyze the conversion of l-arginine to l-ornithine and urea (Fig. 1a). Elevated arginase activity therefore can diminish the bioavailability of l-arginine by substrate competition and decrease NO production, which can lead to endothelial dysfunction [9, 10] and eventually result in adverse cardiovascular issues [11]. An additional level of regulation in NO production is mediated by methylated arginine products l-*N*^G^-monomethyl arginine (MMA), asymmetric dimethylarginine (ADMA), and symmetric dimethylarginine (SDMA) (Fig. 1b). MMA and ADMA are potent endogenous inhibitors of NOS, whereas SDMA inhibits NO production mainly by blocking the cellular uptake of l-arginine [12]. The role of elevated ADMA in inducing endothelial dysfunction has been studied extensively [13–15]; however, the role of SDMA in CVD pathogenesis is not well understood. Importantly, our group and others have demonstrated that elevated plasma levels of both ADMA and SDMA are associated with increased risk for CVD in the general population [14, 16–19].

Despite emerging data showing a relationship between specific l-arginine metabolites and CVD in respective RA cohorts, a comprehensive study evaluating the arginine metabolome in a single RA cohort has not been performed. In the present study, we studied a panel of plasma l-arginine metabolites representing NO metabolism and plasma arginase activity in patients with RA compared with age- and sex-matched healthy control subjects. In addition, we examined associations of CVD risk factors in RA with (a) l-arginine metabolites; (b) global arginine bioavailability ratio (GABR; the ratio of l-arginine to l-ornithine + l-citrulline), which reflects overall status of l-arginine catabolism [20]; and (c) arginine methylation index (ArgMI). ArgMI is an overall gauge for posttranslational methylation of arginine (i.e., ADMA + SDMA/MMA), which we found to be potentially a better predictor of CVD than free methylated arginines [17, 20].

## Methods

### Patient cohort

In our cross-sectional study, patients with RA diagnosed by a board-certified rheumatologist were sequentially selected from the Cleveland Clinic Department of Rheumatic and Immunologic Diseases outpatient rheumatology clinics. Plasma samples were obtained under a protocol approved by the institutional review board (IRB) of the Cleveland Clinic, and all participants gave written informed consent. Associated clinical data and standard of care laboratory values were collected from the patient’s medical records into a de-identified, IRB-approved biospecimen registry. Clinical information collected on the RA disease cohort included body mass index; RA disease duration; RA disease activity (Disease Activity Score in 28 joints [DAS28]); seropositivity status; disease-modifying antirheumatic drug treatment; and CV risk factors, including history of diabetes mellitus, systolic hypertension, dyslipidemia, smoking, and prior history of CVD (defined as myocardial infarction, stroke, coronary artery disease, congestive heart failure, or valvular disease and replacement). Laboratory data included C-reactive protein (CRP) and erythrocyte sedimentation rate (ESR) values. Patients were determined to be seropositive on the basis of a rheumatoid factor > 20 IU/ml or cyclic citrullinated peptide autoantibody level > 20 U.

### Measurement of l-arginine and l-arginine derivatives

Plasma samples from 119 nonfasting patients with RA and 238 nonfasting control subjects were obtained under a protocol approved by the Cleveland Clinic IRB. Plasma aliquots were isolated from whole blood collected in ethylenediaminetetraacetic acid-containing tubes that maintained at 0 °C to 4 °C immediately after phlebotomy, processed within 4 hours of blood draw, and stored at − 80 °C until use. Plasma concentrations of l-arginine, its metabolites (l-ornithine and l-citrulline), and methylated arginine byproducts (MMA, ADMA, and SDMA) were quantified as described in an earlier publication [17]. Briefly, 4 vol of methanol-containing, isotope-labeled internal standards were added to 1 vol of plasma to precipitate protein. The supernatant after centrifugation was analyzed by injection onto a silica column interfaced with an API 4000 Q-TRAP mass spectrometer (AB SCIEX, Framingham, MA, USA). A discontinuous gradient was generated to resolve the analytes by mixing solvent A (0.1% propionic acid in water) with solvent B (0.1% acetic acid in methanol) [21]. Analytes and the isotope-labeled internal standards were monitored by positive multiple reaction mode MS using characteristic precursor–product ion transitions. The parameters for the ion monitoring were optimized for each analyte. Various concentrations of analytes were titrated with control plasma sample to prepare the calibration curves.

### Plasma arginase activity

We measured plasma arginase activity in 119 patients with RA and compared it with that of 148 age- and sex-matched control subjects. Plasma arginase activity was measured using the QuantiChrom Arginase Assay Kit (BioAssay Systems, Hayward, CA, USA) according to the manufacturer’s instructions. Briefly, 5 μl of the plasma was diluted to 40 μl with deionized water (1:8 sample dilution), or 40 μl of deionized water (blank) was treated with kit-provided substrate containing reaction mixture, and then incubated at 37 °C for 2 hours. Arginase-catalyzed urea was measured by colorimetry after adding kit-provided reagents. The optical density was measured at 430 nm. Urea (1 mM) was used as the standard. Arginase activity (expressed as U/L of sample) was calculated. One unit of arginase converts 1 μmol of l-arginine to ornithine and urea per minute at pH 9.5 and 37 °C.

### Statistical analysis

Descriptive summaries of demographic and clinical variables for patients with RA are provided. These include sex, medical history, medications, RA disease activity measures, CVD risk assessments, and laboratory parameters such as traditional systemic inflammatory markers. Categorical variables were compared using Pearson’s chi-square test or Fisher’s exact test, and continuous variables were compared using the *t* test, analysis of variance, or their nonparametric analogues, as appropriate based on distributional assumptions.

Plasma levels of l-arginine and its derivatives are reported for subjects with RA and age- and sex-matched healthy control subjects by means and SDs or by medians within IQRs, as appropriate (primarily based on normalized vs. skewed distribution of the data, respectively). Multivariable logistic regression models are used to estimate the ORs associated with various l-arginine metabolites among RA and control subjects. Correlations between l-arginine, its derivatives’ levels, and a broad range of arthritic, inflammatory, and CV parameters were assessed using Spearman’s correlation. *p* < 0.05 was considered statistically significant. All statistical analyses were performed using R version 3.1.0 (R Core Team, Vienna, Austria).

## Results

### Study population

Our cohort consisted of 119 patients with RA (84% female, mean age 60.6 ± 13.4 yr) and 238 control subjects (82% female, mean age 59 ± 13.9 yr). A subgroup of patients with RA (*n* = 33, 27.7%) from this cohort had a DAS28 assessment at the time of sampling with a median DAS28 score of 2.7 ± 1.2, 2.6 (1.6–3.5). Mean disease duration was 11.7 ± 9.6 years with low median CRP (mg/dl) of 1.2 ± 2.6, 0.4(0.2–0.9) and ESR (mm/h) of 21.3 ± 18.3, 14.5 (7–29.2) (Table 1). This patient population also had a significant history of CVD risk factors, including diabetes, dyslipidemia, and hypertension at 18%, 47%, and 60%, respectively. Patients with a prior history of CVD represented 14% of the population.

**Table 1:** Clinical characteristics of subjects with rheumatoid arthritis (*n* = 119)

Demographics	Values
Demographics	
Male sex	19 (16%)
Age, years	60.6 ± 13.4, 62.0 (53.5–70.5)
BMI, kg/m^2^	28.8 ± 6.3, 28.0 (25–32)
Disease activity	
Seropositive RA**	85 (71.4%)
RF+ (≥ 20)	75 (63.0%)
CCP+ (≥ 20)	59 (49.6%)
Disease duration, yr	11.7 ± 9.6, 9.0 (5–16)
DAS28	2.7 ± 1.2, 2.6 (1.6–3.5)
CV burden assessments	
Diabetes mellitus	21 (17.6%)
Hypertension	71 (59.7%)
Dyslipidemia	56 (47.1%)
Prior CV disease history	17 (14.3%)
Smoking (current)	57 (47.9%)
Medications	
Statin use	32 (26.9%)
Steroid use	54 (45.4%)
Methotrexate use	64 (53.8%)
Biologic DMARD use^a^	61 (51.3%)
Not currently receiving DMARDs	11 (9.2%)
Antihypertensive drugs^b^	55 (77.5%)
Diuretic	29 (40.8%)
Calcium channel blocker	21 (29.6%)
ACE inhibitor	19 (26.8%)
β-Blocker	18 (25.4%)
Angiotensin II receptor blockers	13 (18.3%)
Vasodilator	1 (1.4%)
α_2_-Adrenergic agonist	1 (1.4%)
Laboratory examination results	
ESR, mm/h	21.3 ± 18.3, 14.5 (7–29.2)
> 15 mm/h	45 (37.8%)
≤ 15 mm/h	47 (39.5%)
N/A	27 (22.7%)
CRP (mg/dl)	1.2 ± 2.6, 0.4 (0.2–0.9)
> 1 mg/dl	22 (18.5%)
≤ 1 mg/dl	76 (63.9%)
N/A	21 (17.6%)

*Abbreviations: BMI* Body mass index, *RA* Rheumatoid arthritis, *RF* Rheumatoid factor, *CCP* Cyclic citrullinated peptide, *DAS28* Disease Activity Score in 28 joints, *CV* Cardiovascular, *DMARD* Disease-modifying antirheumatic drug, *ACE* Angiotensin-converting enzyme, *ESR* Erythrocyte sedimentation rate, *CRP* C-reactive protein, *N/A* Not available

Values are given as number (%), mean ± SD, or median (IQR)

^a^ Current use at the time of sampling

^b^ Some patients overlap in multiple subcategories

### Aberrant l-arginine metabolism in subjects with RA

**Fig. 2:**
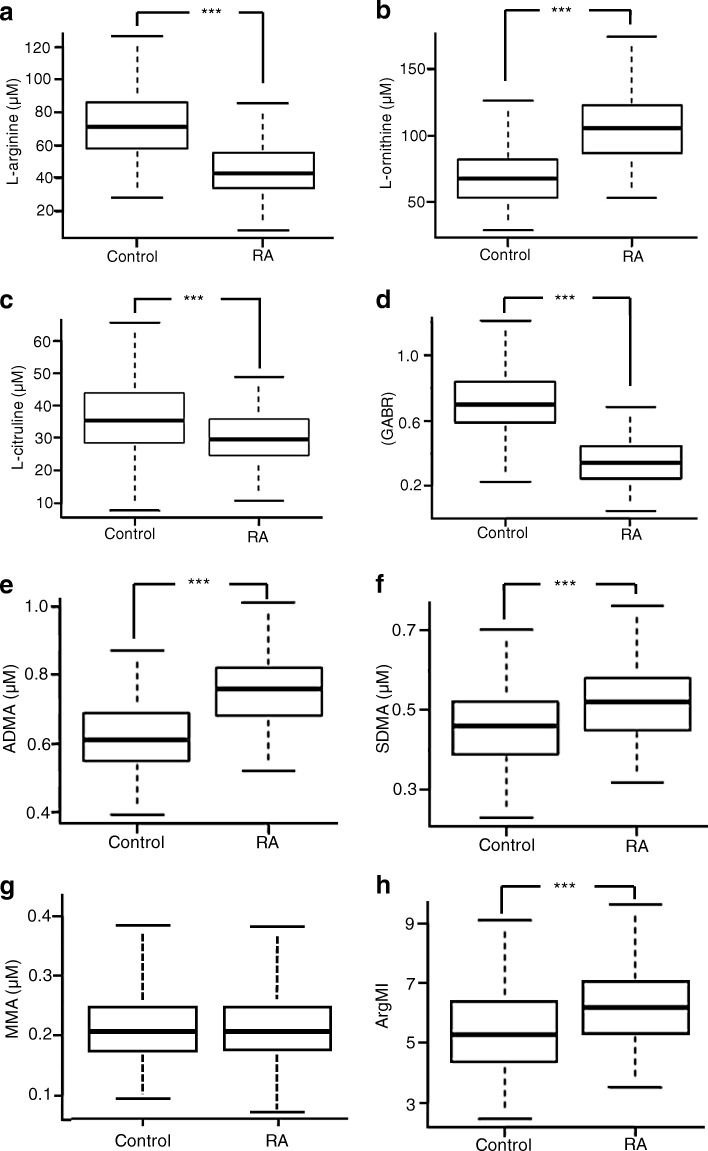
Quantification of l-arginine and l-arginine metabolites in human plasma. Plasma levels of l-arginine and l-arginine catabolic products and methylated arginine derivatives in patients with rheumatoid arthritis (*n* = 119) vs. control subjects (*n* = 238) were measured using LC-MS/MS: l-arginine (**a**), l-ornithine (**b**), l-citrulline (**c**), GABR (**d**), ADMA (**e**), SDMA (**f**), MMA (**g**), and ArgMI (**h**). *GABR* Global arginine bioavailability ratio (i.e., ratio of l-arginine to l-ornithine + l-citrulline), *ADMA* Asymmetric dimethylarginine, *SDMA* Symmetric dimethylarginine, *ArgMI* Arginine methylation index (i.e., ADMA + SDMA/MMA), *MMA* l-*N*^G^-monomethyl arginine, *RA* Rheumatoid arthritis. *** *p* < 0.001

We compared the l-arginine, l-arginine catabolic products, and methylated arginine derivatives in patients with RA and age- and sex-matched control subjects (Fig. 2). Compared with control subjects, the RA cohort had significantly lower levels of l-arginine (43.2 vs. 71.7 μmol/L, *p* < 0.001) (Fig. 2a) and GABR (0.34 vs. 0.70, *p* < 0.001) (Fig. 2d). The RA cohort also showed a concomitant increase in the arginase catabolic product l-ornithine (106.3 vs. 67.6 μmol/L, *p* < 0.001) (Fig. 2b) and diminished levels of the NOS catabolic product l-citrulline (29.6 vs. 35.6 μmol/L, *p* < 0.001) (Fig. 2c). In the RA cohort, we also found elevated levels of ADMA (0.76 vs. 0.61 μmol/L, *p* < 0.001) (Fig. 2e), SDMA (0.52 vs. 0.46 μmol/L, *p* < 0.001) (Fig. 2f), and the index of arginine methylation, ArgMI [(ADMA+SDMA)/MMA] (6.2 vs. 5.30, *p* < 0.001) (Fig. 2h). Compared with control subjects, plasma level of MMA did not change significantly in patients with RA (0.21 μmol/L in control and RA) (Fig. 2g).

Next, we performed multivariable logistic regression analysis to estimate the ORs associated with plasma l-arginine metabolites among the patients with RA. After adjustment for decreased renal function, which influences steady-state level of plasma SDMA in particular [22], we found that ADMA, SDMA, and ArgMI each had a positive relationship with RA with ORs >1 (Table 2): ADMA (3.82 [95% CI, 2.67–5.46], *p* < 0.001), SDMA (1.43 [95% CI, 1.07–1.93], *p* = 0.0163), and ArgMI (2.0 [95% CI, 1.50–2.65], *p* < 0.001). GABR (OR, 0.03 [95% CI, 0.01–0.07], *p* < 0.001) and l-arginine (OR, 0.12 [95% CI, 0.07–0.21], *p* < 0.001) each showed a negative relationship with RA. In the general population; both GABR and ArgMI have been shown to be better predictors of major adverse cardiac events than free l-arginine or individual methylated arginine derivatives [20, 23]_._

**Table 2:** Elevated l-arginine metabolites in plasma are associated with rheumatoid arthritis incidence

	OR (95% CI)	*p* Value
ADMA		
Unadjusted OR	3.79 (2.73–5.26)	< 0.001
Adjusted OR	3.82 (2.67–5.46)	< 0.001
SDMA		
Unadjusted OR	1.68 (1.31–2.16)	< 0.001
Adjusted OR	1.43 (1.07–1.93)	0.0163
ArgMI		
Unadjusted OR	2.03 (1.55–2.65)	< 0.001
Adjusted OR	2 (1.5–2.65)	< 0.001
GABR		
Unadjusted OR	0.04 (0.02–0.08)	< 0.001
Adjusted OR	0.03 (0.01–0.07)	< 0.001
l-arginine		
Unadjusted OR	0.14 (0.09–0.22)	< 0.001
Adjusted OR	0.12 (0.07–0.21)	< 0.001
l-ornithine		
Unadjusted OR	7.36 (4.76–11.39)	< 0.001
Adjusted OR	7.56 (4.68–12.22)	< 0.001
l-citrulline		
Unadjusted OR	0.55 (0.42–0.71)	< 0.001
Adjusted OR	0.38 (0.27–0.53)	< 0.001

*Abbreviations: ADMA* Asymmetric dimethylarginine, *SDMA* Symmetric dimethylarginine, *ArgMI* Arginine methylation index (i.e., ADMA + SDMA/l-*N*^G^-monomethyl arginine), *GABR* Global arginine bioavailability ratio (i.e., ratio of l-arginine to l-ornithine + l-citrulline)

Adjusted for age, sex, and decreased renal function (creatinine > 1.4 mg/dl or GFR ≤ 60 ml/min/1.73 m^2^. ORs are presented per SD

### SDMA is associated with hypertension and hyperlipidemia in subjects with RA

We further determined whether the levels of ADMA, SDMA, ArgMI, and GABR were associated with CVD risk factors in patients with RA (Table 3). Among various CVD risk factors, patients with RA with a prior history of hypertension (*n* = 71) showed statistically significant elevated levels of SDMA (μmol/L plasma) compared with patients with RA with normal blood pressure (*n* = 48): (0.6 ± 0.2, 0.5 [0.5–0.6]; vs. 0.5 ± 0.1, 0.5 [0.4–0.5]; *p* = 0.006). We also found a negative association of SDMA and ArgMI with seropositivity. Plasma SDMA level in seropositive patients with RA (*n* = 85) compared with seronegative patients (*n* = 34) were as follows: 0.5 ± 0.1, 0.5 (0.4–0.6) vs. 0.6 ± 0.2, 0.5 (0.5–0.6) (*p* = 0.035). Similarly, we found a lower ArgMI in seropositive patients compared with seronegative patients: 6.2 ± 1.4, 6.0 (5.3–6.9) vs. 7.6 ± 2.9, 7.3 (6.0–8.8) (*p* = 0.03).

**Table 3:** Comparison of plasma levels of l-arginine metabolites in patients with rheumatoid arthritis with and without cardiovascular risk factors

CV risk factors		ADMA (μmol/L)		SDMA (μmol/L)		ArgMI		GABR	
	No. of patients	Value	*p* Value	Value	*p* Value	Value	*p* Value	Value	*p* Value
History of diabetes (+)	98	0.8 ± 0.1 0.8 (0.7–0.8)	0.484	0.5 ± 0.1 0.5 (0.4–0.6)	0.652	6.6 ± 1.9 6.3 (5.4–7.3)	0.031	0.4 ± 0.2 0.3 (0.2–0.4)	0.556
History of diabetes (−)	21	0.8 ± 0.1 0.8 (0.7–0.9)		0.5 ± 0.1 0.5 (0.4–0.6)		5.9 ± 1.3 6.1 (5–6.4)		0.4 ± 0.2, 0.4 (0.3–0.5)	
History of hyperlipidemia (−)	63	0.8 ± 0.1 0.8 (0.7–0.8)	0.260	0.5 ± 0.1 0.5 (0.4–0.6)	0.054	6.5 ± 2.2 6.1 (5.3–7.1)	0.850	0.4 ± 0.2 0.4 (0.3–0.5)	0.259
History of hyperlipidemia (+)	56	0.7 ± 0.1 0.8 (0.7–0.8)		0.6 ± 0.2 0.5 (0.4–0.6)		6.5 ± 1.4 6.5 (5.7–7)		0.3 ± 0.2 0.3 (0.2–0.4)	
History of HTN (−)	48	0.8 ± 0.1 0.7 (0.7–0.8)	0.662	0.5 ± 0.1 0.5 (0.4–0.5)	0.006*	6.3 ± 1.7 6 (5.3–7.4)	0.346	0.4 ± 0.1 0.3 (0.2–0.4)	0.875
History of HTN (+)	71	0.8 ± 0.1 0.8 (0.7–0.8)		0.6 ± 0.2 0.5 (0.5–0.6)		6.6 ± 2 6.4 (5.7–7)		0.4 ± 0.2 0.3 (0.3–0.4)	
Two or more CVD risk factors	46	0.8 ± 0.1 0.8 (0.7–0.8)	0.640	0.6 ± 0.2 0.5 (0.5–0.6)	0.108	6.5 ± 1.4 6.4 (5.8–6.9)	0.986	0.4 ± 0.2 0.3 (0.3–0.4)	0.827
Less than two CVD risk factors	73	0.8 ± 0.1 0.8 (0.7–0.8)		0.5 ± 0.1 0.5 (0.4–0.6)		6.5 ± 2.1 6.1 (5.3–7.3)		0.4 ± 0.2 0.3 (0.2–0.5)	
Smoking (−)	62	0.8 ± 0.1 0.7 (0.7–0.8)	0.313	0.5 ± 0.1 0.5 (0.4–0.6)	0.754	6.5 ± 1.6 6.2 (5.5–7)	0.912	0.4 ± 0.2 0.3 (0.2–0.4)	0.968
Smoking (+)	57	0.8 ± 0.1 0.8 (0.7–0.8)		0.5 ± 0.2 0.5 (0.4–0.6)		6.5 ± 2.2 6.1 (5.1–7.2)		0.4 ± 0.2 0.3 (0.2–0.4)	
History of CVD (−)	101	0.8 ± 0.1 0.8 (0.7–0.8)	0.542	0.5 ± 0.1 0.5 (0.4–0.6)	0.379	6.6 ± 1.9 6.2 (5.4–7.3)	0.270	0.4 ± 0.2 0.3 (0.2–0.4)	0.473
History of CVD (+)	17	0.8 ± 0.1 0.8 (0.7–0.8)		0.5 ± 0.1 0.5(0.4–0.6)		6.2 ± 1.2 6.4 (5–6.7)		0.4 ± 0.2 0.4 (0.2–0.6)	
Seropositive (+)	85	0.8 ± 0.1 0.8 (0.7–0.8)	0.293	0.5 ± 0.1 0.5 (0.4–0.6)	0.035	6.2 ± 1.4 6 (5.3–6.9)	0.03	0.4 ± 0.2 0.3 (0.3–0.4)	0.434
Seronegative (−)	34	0.7 ± 0.1 0.7 (0.7–0.8)		0.6 ± 0.2 0.5 (0.5–0.6)		7.6 ± 2.9 7.3 (6–8.8)		0.4 ± 0.2 0.4 (0.3–0.5)	

*Abbreviations: ADMA* Asymmetric dimethylarginine, *SDMA* Symmetric dimethylarginine, *ArgMI* Arginine methylation index (i.e., ADMA + SDMA/l-*N*^G^-monomethyl arginine), *GABR* Global arginine bioavailability ratio (i.e., ratio of l-arginine to l-ornithine + l-citrulline), *CV* Cardiovascular, *CVD* Cardiovascular disease, *HTN* Hypertension

Further, as shown in Table 4, patients in the highest SDMA quartile (≥ 0.58 μmol/L) had a higher prevalence of the following cardiovascular risk factors than those in the lowest quartile (< 0.44 μmol/L): hypertension (78.8% vs. 44.8%, *p* = 0.039), hyperlipidemia (63.6% vs. 31%, *p* = 0.014) and two or more CV risk factors (54.5% vs. 27.6%, *p* = 0.022). Disease duration, DAS28 scores, and inflammatory markers CRP and ESR were not associated with ADMA, SDMA, ArgMI, or GABR levels (data not shown).

**Table 4:** Prevalence of hypertension and hyperlipidemia in patients with rheumatoid arthritis in highest symmetric dimethylarginine quartile

SDMA Quartiles					
	Quartile 1	Quartile 2	Quartile 3	Quartile 4	*p* Value
No. of patients	29	29	28	33	
SDMA, μM/L	< 0.44	0.45–0.51	0.52–0.57	≥ 0.58	
Demographics					
Age, yr	52.8 ± 12.4	59.7 ± 15.4	62.5 ± 10.9	66.7 ± 11.2	< 0.001^a^
BMI, kg/m^2^	29.24 ± 6.83	27.55 ± 4.79	28.55 ± 8.2	29.67 ± 5.38	0.592
Disease activity					
Disease duration, yr	11.19 ± 9.91	13 ± 10.14	10.48 ± 7.05	11.96 ± 11.02	0.805
DAS28	3.47 (2.51–3.9)	2.06 (1.38–2.87)	2.42 (1.71–2.91)	2.76 (1.82–3.63)	0.117
CV burden and assessments, *n* (%)					
History of diabetes,	5 (17.24%)	7 (24.14%)	3 (10.71%)	6 (18.18%)	0.621
History of hyperlipidemia	9 (31%)	17 (58.6%)	9 (32.1%)	21 (63.6%)	0.014^a^
History of hypertension	13 (44.8%)	17 (58.6%)	15 (53.6%)	26 (78.8%)	0.039^a^
Two or more CV risk factors	8 (27.6%)	14 (48.3%)	6 (21.4%)	18 (54.5%)	0.022^a^
History of CVD	4 (13.8%)	2 (6.9%)	5 (17.9%)	6 (18.2%)	0.585
Smoking	14 (48.3%)	12 (41.4%)	11 (39.3%)	20 (60.6%)	0.330
Laboratory examination results					
ESR, mm/h	14.5 (8.25–28.75)	11 (7–30)	10 (7–24)	21 (9–28.75)	0.944
CRP, mg/dl	0.6 (0.2–1.7)	0.3 (0.2–0.6)	0.4 (0.1–1)	0.6 (0.2–1.6)	0.678

*Abbreviations: SDMA* Symmetric dimethylarginine, *BMI* Body mass index, *DAS28* Disease Activity Score in 28 joints, *CV* Cardiovascular, *CVD* Cardiovascular disease, *ESR* Erythrocyte sedimentation rate, *CRP* C-reactive protein

^a^ statistically significant

### Arginase activity is elevated in RA

**Fig. 3:**
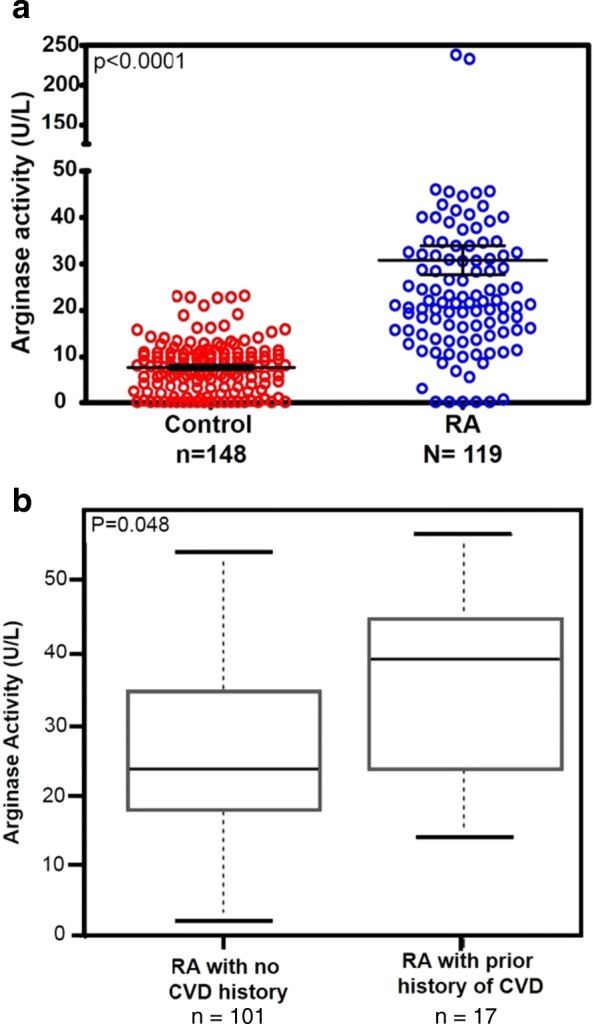
**a** Plasma arginase activity in patients with rheumatoid arthritis (RA) vs. non-RA control subjects. Plasma arginase activity was measured using QuantiChrom Arginase Assay Kit. One unit of arginase converts 1 μmol of l-arginine to ornithine and urea per minute at pH 9.5 and 37 °C. **b** Elevated arginase activity is associated with RA with prior history of cardiovascular disease (CVD). *p* = 0.048

We measured plasma arginase activity in 119 patients with RA (Table 1) and compared it with that of 148 age- and sex-matched control subjects. These control subjects had no RA disease activity; however, other clinical parameters of the control subjects were not evaluated. The plasma arginase activity was significantly elevated in the RA cohort (> 400%) compared with control group (*p* < 0.0001) (Fig. 3a).

Further analysis showed heightened arginase activity in a subgroup of 17 patients with RA with existing CVD compared with patients with RA without CVD (*n* = 101) (*p* = 0.048) (Fig. 3b). Interestingly, elevated arginase activity did not show an association with traditional risk factors such as hypertension, type 2 diabetes, dyslipidemia, and smoking.

## Discussion

Using a comprehensive metabolomic analysis, we tested whether plasma l-arginine metabolites representing l-arginine bioavailability and l-arginine metabolites reflecting NO metabolism were altered in an RA cohort. Compared with healthy control subjects, our RA cohort showed robust increases in the arginase catabolic product l-ornithine (~ 60% increase) and diminished NOS catabolic product l-citrulline (~ 18% decrease), with a significant decrease in arginine bioavailability (*p* < 0.001). We also found a 400% increase in plasma arginase activity in patients with RA compared with control subjects.

Several clinical studies have shown that increased arginase activity is associated with endothelial dysfunction in the general population [11]; however, less is known about the RA population. Arginases are localized in the cytoplasm or in the mitochondria of various cell types, including endothelial cells and immune cells, in particular monocytes/macrophages [24, 25]. Systemic Inflammatory conditions can increase arginase expression in these cell types [26]. It is possible that a higher turnover of these cells can cause elevated arginase levels that can be detected in the blood. Arginases are also present in erythrocytes [27, 28], and perturbation of erythrocytes can release arginases into the circulation. One relatively small study showed that serum arginase activity and arginase protein levels were elevated in patients with RA (*n* = 25) [29]. Our larger study comparing 119 patients with RA and 148 age- and sex-matched healthy control subjects showed a robust increase in arginase activity along with a reduced level of its substrate, l-arginine, in the RA plasma.

Our analysis shows no association between increased arginase activity and RA disease activity (DAS28 score). This could be due to a relatively low RA disease activity in our cohort (median DAS28 score, 2.7 [1.6–3.5] (Table 1). Alternatively, arginase activity may not elicit a significant effect on the RA pathogenesis per se, but may influence the induction of subclinical endothelial dysfunction in patients with RA. In support of this hypothesis, Prati et al. showed that a specific arginase inhibitor restores endothelial function without ameliorating disease activity in an acute rat model of arthritis [30]. Interestingly, the disconnect was further supported by our observation that seronegative subjects had statistically significant elevated levels of SDMA and ArgMI compared with seropositive patients (Table 3), although the latter showed higher extraarticular disease manifestations. The disconnect between arginase activity and RA disease activity could have clinical implications because arginase activity can be a biomarker of increased CVD risk independent of the patient’s disease state.

Negative regulation of NO synthesis can also be mediated via overproduction of methylated arginine analogues such as MMA, ADMA, and SDMA. Among the methylated derivatives, ADMA, a potent endogenous inhibitor of NOS and a marker of endothelial dysfunction, has been shown by our group and others to serve as an independent risk factor for cardiovascular events in the general population [14, 16]. Multiple studies show that ADMA levels were significantly elevated in patients with RA compared with control subjects [31–34]. However, Sandoo et al, showed a lack of association microvascular and macrovascular endothelial function in patients with rheumatoid arthritis [35]. Further, a recent study showed that ADMA levels were inversely correlated with flow-mediated dilation in patients with RA [15]. Erre et al. showed that ADMA is not associated with arterial stiffness in patients with RA [36]. These findings are in agreement with another study that showed supplementation of tetrahydrobiopterin, a cofactor for the production of NO, improved endothelial function but failed to improve aortic stiffness [37]. Our study shows that plasma ADMA is significantly elevated in patients with RA compared with control subjects; in future larger studies, we will include endothelial function and determine the relationship of ADMA/SDMA and endothelial function in an RA group.

In our study, levels of SDMA were also significantly elevated in patients with RA (*p* < 0.001). An elevated SDMA in patients with RA level is associated with an adjusted OR of 1.43 (1.07–1.93, *p* = 0.0163). However, one group showed a decrease in SDMA level in patients with RA compared with control patients and an inverse correlation between SDMA level and microvascular function [38, 39]. The same group also showed that SDMA levels in patients with RA were independent of cumulative inflammatory burden and that there was no association with cardiovascular risk factors, including hypertension [40]. This discrepancy in comparative levels of SDMA in patients with RA between the two studies may be due to multiple factors, including differences in subject age, disease activity, and extent of comorbid renal disease, as well as, more important, divergent techniques used in the measurement of SDMA in the plasma.

Although ADMA is emerging as a biomarker of CVD, the implications of elevated circulating SDMA are not well understood. Recent reports, including our group’s, have shown an association between elevated plasma SDMA levels and higher mortality in patients with CVD [17–19]. It has been shown that association of SDMA with CVD risk factors, in particular with hypertension, may arise from compromised renal function [17–19], given that SDMA is eliminated by renal excretion and has been shown to be a marker of estimated glomerular filtration rate [22]. However, multivariable logistic regression analysis (Table 3), adjusted for glomerular filtration rate and plasma creatinine level, demonstrated that elevated SDMA was associated with hypertension in the RA cohort independent of renal function. SDMA interquartile analysis (Table 4) further demonstrated that patients in the highest SDMA quartile had a significantly higher prevalence of hypertension and hyperlipidemia than those in the lowest SDMA quartile. ADMA, ArgMI, and GABR, which have recently emerged as candidate biomarkers of CV risk [14, 16, 18, 20, 23], failed to show significant changes in any of the traditional CVD risk factors analyzed. Our results suggest that SDMA potentially functions as a biomarker of cardiovascular risk factors in RA. More studies are needed to gain an understanding of the underlying mechanisms that link SDMA specifically to hypertension and hyperlipidemia in patients with RA.

### Study limitations

One limitation of this study is the relatively small sample size, which may limit the statistical power of the conclusions. We did not evaluate the population characteristics, other than age, sex, and disease activity, in the control population. Also, this was a single-center study using a cross-sectional evaluation of biomarkers. Because this study did not use fasting blood samples, it is possible that plasma levels of arginine and its metabolic products were influenced by food intake. Further, medical interventions that might have the potential to alter plasma l-arginine metabolites were not considered. No direct physiologic vascular measures were taken to directly link dysfunctional arginine metabolism to vascular functional changes and potential cardiovascular risks. We also acknowledge that, other than arginases and NOS, levels of l-arginine, l-citrulline, and l-ornithine can be altered by the aberrant release/uptake of these molecules in and out of the circulation and dysregulation of enzymes that participate in their biosynthesis [10]. We did not measure these parameters in our study. Nevertheless, our studies point to the importance of understanding NO synthesis-related dysfunctional l-arginine metabolic pathways in RA that may provide novel therapeutic and prophylactic approaches to improve vascular health and thereby reduce CVD risk in patients with RA and related rheumatic diseases.

## Conclusions

We performed a comprehensive analysis of plasma l-arginine metabolic products and methylated arginine derivatives in a cohort of patients with RA and control subjects. We identified diminished global l-arginine availability and decreased levels of the NOS catabolic product l-citrulline, whereas levels of both arginase activity and its catabolic product l-ornithine were elevated in plasma of patients with RA. Additionally, we found increased levels of endogenous inhibitors of NO production ADMA and SDMA in the plasma of patients with RA. Further, plasma SDMA levels were associated with cardiovascular risk factors, hypertension, and hyperlipidemia, whereas elevated arginase activity was associated with prior history of CVD in a subgroup of patients with RA. Our study suggests that increased ArgMI and diminished global arginine bioavailability with concomitant elevated arginase activity in plasma can potentially predict CVD risk in patients with RA. Additional controlled longitudinal studies are required to establish the importance of these pathways in the development of atherosclerosis and cardiac diseases in patients with RA.

## Abbreviations

ACE: Angiotensin-converting enzyme
ADMA: Asymmetric dimethylarginine
ArgMI: Arginine methylation index
BMI: Body mass index
CCP: Cyclic citrullinated peptide
CRP: C-reactive protein
CV: Cardiovascular
CVD: Cardiovascular disease
DAS28: Disease Activity Score in 28 joints
DMARD: Disease-modifying antirheumatic drug
ESR: Erythrocyte sedimentation rate
GABR: Global arginine bioavailability ratio
HTN: Hypertension
MMA: l-*N*^G^-monomethyl arginine
NO: Nitric oxide
NOS: Nitric oxide synthase
PRMT: Protein arginine methyltransferase
RA: Rheumatoid arthritis
RF: Rheumatoid factor
SDMA: Symmetric dimethylarginine

## References

[1] Helmick CG, Felson DT, Lawrence RC, Gabriel S, Hirsch R, Kwoh CK, Liang MH, Kremers HM, Mayes MD, Merkel PA (2008). Estimates of the prevalence of arthritis and other rheumatic conditions in the United States. Part I. Arthritis Rheum.

[2] Naz SM, Symmons DP (2007). Mortality in established rheumatoid arthritis. Best Pract Res Clin Rheumatol.

[3] Hunter TM, Boytsov NN, Zhang X, Schroeder K, Michaud K, Araujo AB (2017). Prevalence of rheumatoid arthritis in the United States adult population in healthcare claims databases, 2004-2014. Rheumatol Int.

[4] Aviña-Zubieta JA, Choi HK, Sadatsafavi M, Etminan M, Esdaile JM, Lacaille D (2008). Risk of cardiovascular mortality in patients with rheumatoid arthritis: a meta-analysis of observational studies. Arthritis Rheum.

[5] Choy E, Ganeshalingam K, Semb AG, Szekanecz Z, Nurmohamed M (2014). Cardiovascular risk in rheumatoid arthritis: recent advances in the understanding of the pivotal role of inflammation, risk predictors and the impact of treatment. Rheumatology (Oxford).

[6] Amaya-Amaya J, Sarmiento-Monroy JC, Mantilla RD, Pineda-Tamayo R, Rojas-Villarraga A, Anaya JM (2013). Novel risk factors for cardiovascular disease in rheumatoid arthritis. Immunol Res.

[7] del Rincon ID, Williams K, Stern MP, Freeman GL, Escalante A (2001). High incidence of cardiovascular events in a rheumatoid arthritis cohort not explained by traditional cardiac risk factors. Arthritis Rheum.

[8] Steppan J, Nyhan D, Berkowitz DE (2013). Development of novel arginase inhibitors for therapy of endothelial dysfunction. Front Immunol.

[9] Totoson P, Maguin-Gate K, Prati C, Wendling D, Demougeot C (2014). Mechanisms of endothelial dysfunction in rheumatoid arthritis: lessons from animal studies. Arthritis Res Ther.

[10] Wu G, Morris SM (1998). Arginine metabolism: nitric oxide and beyond. Biochem J.

[11] Durante W, Johnson FK, Johnson RA (2007). Arginase: a critical regulator of nitric oxide synthesis and vascular function. Clin Exp Pharmacol Physiol.

[12] Cardounel AJ, Cui H, Samouilov A, Johnson W, Kearns P, Tsai AL, Berka V, Zweier JL (2007). Evidence for the pathophysiological role of endogenous methylarginines in regulation of endothelial NO production and vascular function. J Biol Chem.

[13] Alderton WK, Cooper CE, Knowles RG (2001). Nitric oxide synthases: structure, function and inhibition. Biochem J.

[14] Bouras G, Deftereos S, Tousoulis D, Giannopoulos G, Chatzis G, Tsounis D, Cleman MW, Stefanadis C (2013). Asymmetric dimethylarginine (ADMA): a promising biomarker for cardiovascular disease?. Curr Top Med Chem.

[15] Senturk T, Yilmaz N, Sargin G, Koseoglu K, Yenisey C (2016). Relationship between asymmetric dimethylarginine and endothelial dysfunction in patients with rheumatoid arthritis. Eur J Rheumatol.

[16] Nicholls SJ, Wang Z, Koeth R, Levison B, DelFraino B, Dzavik V, Griffith OW, Hathaway D, Panza JA, Nissen SE (2007). Metabolic profiling of arginine and nitric oxide pathways predicts hemodynamic abnormalities and mortality in patients with cardiogenic shock after acute myocardial infarction. Circulation.

[17] Wang Z, Tang WH, Cho L, Brennan DM, Hazen SL (2009). Targeted metabolomic evaluation of arginine methylation and cardiovascular risks: potential mechanisms beyond nitric oxide synthase inhibition. Arterioscler Thromb Vasc Biol.

[18] Meinitzer A, Kielstein JT, Pilz S, Drechsler C, Ritz E, Boehm BO, Winkelmann BR, Marz W (2011). Symmetrical and asymmetrical dimethylarginine as predictors for mortality in patients referred for coronary angiography: the Ludwigshafen Risk and Cardiovascular Health study. Clin Chem.

[19] Gore MO, Lüneburg N, Schwedhelm E, Ayers CR, Anderssohn M, Khera A, Atzler D, de Lemos JA, Grant PJ, McGuire DK (2013). Symmetrical dimethylarginine predicts mortality in the general population: observations from the Dallas heart study. Arterioscler Thromb Vasc Biol.

[20] Tang WH, Wang Z, Cho L, Brennan DM, Hazen SL (2009). Diminished global arginine bioavailability and increased arginine catabolism as metabolic profile of increased cardiovascular risk. J Am Coll Cardiol.

[21] Wang Z, Levison BS, Hazen JE, Donahue L, Li XM, Hazen SL (2014). Measurement of trimethylamine-N-oxide by stable isotope dilution liquid chromatography tandem mass spectrometry. Anal Biochem.

[22] Bode-Boger SM, Scalera F, Kielstein JT, Martens-Lobenhoffer J, Breithardt G, Fobker M, Reinecke H (2006). Symmetrical dimethylarginine: a new combined parameter for renal function and extent of coronary artery disease. J Am Soc Nephrol.

[23] Tang WH, Shrestha K, Wang Z, Troughton RW, Klein AL, Hazen SL (2013). Diminished global arginine bioavailability as a metabolic defect in chronic systolic heart failure. J Card Fail.

[24] Durante W (2013). Role of arginase in vessel wall remodeling. Front Immunol.

[25] Ming XF, Rajapakse AG, Yepuri G, Xiong Y, Carvas JM, Ruffieux J, Scerri I, Wu Z, Popp K, Li J (2012). Arginase II promotes macrophage inflammatory responses through mitochondrial reactive oxygen species, contributing to insulin resistance and atherogenesis. J Am Heart Assoc.

[26] Munder M (2009). Arginase: an emerging key player in the mammalian immune system. Br J Pharmacol.

[27] Pernow J, Jung C (2013). Arginase as a potential target in the treatment of cardiovascular disease: reversal of arginine steal?. Cardiovasc Res.

[28] Spector EB, Rice SC, Kern RM, Hendrickson R, Cederbaum SD (1985). Comparison of arginase activity in red blood cells of lower mammals, primates, and man: evolution to high activity in primates. Am J Hum Genet.

[29] Huang LW, Chang KL, Chen CJ, Liu HW (2001). Arginase levels are increased in patients with rheumatoid arthritis. Kaohsiung J Med Sci.

[30] Prati C, Berthelot A, Kantelip B, Wendling D, Demougeot C (2012). Treatment with the arginase inhibitor N_w_-hydroxy-nor-L-arginine restores endothelial function in rat adjuvant-induced arthritis. Arthritis Res Ther.

[31] Turiel M, Atzeni F, Tomasoni L, de Portu S, Delfino L, Bodini BD, Longhi M, Sitia S, Bianchi M, Ferrario P (2009). Non-invasive assessment of coronary flow reserve and ADMA levels: a case-control study of early rheumatoid arthritis patients. Rheumatology (Oxford).

[32] Surdacki A, Martens-Lobenhoffer J, Wloch A, Marewicz E, Rakowski T, Wieczorek-Surdacka E, Dubiel JS, Pryjma J, Bode-Boger SM (2007). Elevated plasma asymmetric dimethyl-l-arginine levels are linked to endothelial progenitor cell depletion and carotid atherosclerosis in rheumatoid arthritis. Arthritis Rheum.

[33] Dimitroulas T, Sandoo A, Kitas GD (2012). Asymmetric dimethylarginine as a surrogate marker of endothelial dysfunction and cardiovascular risk in patients with systemic rheumatic diseases. Int J Mol Sci.

[34] Sandoo A, Dimitroulas T, Hodson J, Smith JP, Douglas KM, Kitas GD (2015). Cumulative inflammation associates with asymmetric dimethylarginine in rheumatoid arthritis: a 6 year follow-up study. Rheumatology (Oxford).

[35] Sandoo A, Dimitroulas T (2012). Veldhuijzen van Zanten JJ, Smith JP, Metsios GS, Nightingale P, Stavropoulos-Kalinoglou A, Kitas GD: Lack of association between asymmetric dimethylarginine and in vivo microvascular and macrovascular endothelial function in patients with rheumatoid arthritis. Clin Exp Rheumatol.

[36] Erre GL, Piras A, Mura S, Mundula N, Piras M, Taras L, Longu MG, Saba PS, Ganau A, Carru C (2016). Asymmetric dimethylarginine and arterial stiffness in patients with rheumatoid arthritis: a case-control study. J Int Med Res.

[37] Mäki-Petäjä KM, Day L, Cheriyan J, Hall FC, Östör AJ, Shenker N, Wilkinson IB (2016). Tetrahydrobiopterin supplementation improves endothelial function but does not alter aortic stiffness in patients with rheumatoid arthritis. J Am Heart Assoc.

[38] Dimitroulas T, Hodson J, Sandoo A, Smith J, Kitas GD (2015). Symmetric dimethylarginine (SDMA) serum levels in rheumatoid arthritis: correlations with insulin resistance and disease activity scores. Amino Acids.

[39] Dimitroulas T, Hodson J, Sandoo A, Smith J, Kitas GD (2017). Endothelial injury in rheumatoid arthritis: a crosstalk between dimethylarginines and systemic inflammation. Arthritis Res Ther.

[40] Dimitroulas T, Hodson J, Sandoo A, Smith JP, Douglas KM, Kitas GD (2015). Symmetric dimethylarginine is not associated with cumulative inflammatory load or classical cardiovascular risk factors in rheumatoid arthritis: a 6-year follow-up study. Mediat Inflamm.

